# Spinal Irritation

**Published:** 1870-04

**Authors:** 


### Spinal Irritation.

Dr. William A. Hammond read, in January last, before the Medical Society of the County of New York, a very valuable paper upon *Spinal Irritation*, which appears in the *Psychological Journal* for April, and should be read by every physician. The editor of the *New York Medical Journal* thus gives, in brief, some of its chief points:

After alluding to the distrust experienced by several distinguished writers, and shared by a large part of the profession, as to the existence of an independent affection which may properly be called "spinal irritation," the speaker stated emphatically his own conviction that there is such a disorder of the spinal cord, by no means rare or unimportant, and no more to be confounded with hysteria, or the various other affections which it may simulate, and to which its symptoms have so often been referred, than with organic disease of the cord. He carefully reviewed the literature of the subject, giving a *resume* of the most important facts and opinions advanced concerning it, from the introduction of the term by Dr. C. Brown, of Glasglow, in 1828, down to the present time. He then gave his own views, based upon a study of one hundred and twelve cases occurring in his private practice, eighty-three of which had been fully recorded, and twenty-nine less completely.

*Symptoms.*—(*a*) Centric symptoms: 1. Tendernesss at one or more points over the spinal column, increased by pressure. This symptom Dr. H. regards as invariably present, although sometimes developed only by careful examination, and occasionally appearing only several moments after the pressure is applied. Any case which does not exhibit it he excludes from the category of spinal irritation. Dr. Austin Flint and some others think it may be absent in exceptional cases. The tenderness varies in character from a dull ache, seated in the deeper tissues and developed by strong pressure, to a lancinating pain, seated in the skin and subcutaneous areolar tissue, and excited by slight pressure. It varies in degree from a slight discomfort to a hyperæsthesia rendering the touch of the clothing insufferable. It may be limited in extent to the spot under pressure, or the pain may be propagated along the spinal nerve. The seat of the tenderness is most frequently the dorsal region, but may be the cervical or the lumbar, and it may extend over the whole spine. Each location has its characteristic eccentric symptoms. 2. Pain in the cord. The tenderness above described was external to the vertebral canal. The pain now spoken of is in the cord itself, and therefore cannot be excited (unless in a reflex way, through the former) by pressure on the spinous processes; but it may be excited by percussion and by motion of the spinal column. The pain is commonly felt near the point of external tenderness, but may be distant from it. It was present in one hundred and nine of Dr. Hammond's cases.

(*b*) Eccentric symptoms. These constitute the most noticeable ones, and vary in accordance with the part of the cord irritated. Among those occurring in Dr. Hammond's cases are the following, under their respective regions: 1. *Cervical irritation:* Vertigo, headache, tinnitus aurium, visual disturbance, sense of frontal constriction; tenderness of scalp; mental aberration (more or less marked in every case); insomnia, or excessive somnolence; neuralgic pains and motor disturbance in parts deriving their nervous supply from the affected region—in scalp and face, if this were the upper cervical; in upper part of chest and upper extremities, if it were the lower cervical; nausea and vomiting, but *not* gastric pain. 2. *Dorsal irritation:* Gastralgia (in every case), gastric flatulence, acidity, nausea and vomiting, pyrosis; palpitation, cardiac oppression, syncope; dyspnœa, cough; intercostal neuralgia, infra-mammary pain (very frequent); motor disorder (spasm or paralysis). 2. *Lumbar irritation:* Neuralgia of lower extremities, and sometimes of back and abdomen; uterine, ovarian, and rectal pain; strangury; tonic spasm of muscles in lower extremities, clonic spasms (occasional in every case), paralysis. The above symptoms are taken from cases where the tenderness was located in but a single region of the spine. In those where it was located in both the cervical and the dorsal region, or both the dorsal and the lumbar, the symptoms presented a combination of those characteristic of each region; and in ten cases, where the whole spine was tender, they were quite irregular in their manifestations from time to time.

*Causes.*—Sex is the strongest predisposing cause, ninety-three of the one-hundred and twelve cases being females. Age has its effect, fifty of eighty-three cases being between fifteen and twenty-five years. Hereditary influence was ascertained in some instances. The exciting causes are often impossible to fix. Among those determined in the cases reported, are mechanical violence, sexual excesses, mental fatigue and anxiety, innutrition, abuse of alcohol and of opium, exhausting diseases

*Pathology.*—The essential pathological condition in this affection Dr. Hammond considers to be anæmia of the cord, and he gives *in extenso* his reasons for this opinion. Other writers have attributed the spinal irritation to congestion, inflammation, and many other conditions. That anæmia may cause the irritability, is shown by analogy, and that local anæmias may be produced is explicable with our present knowledge of the vaso-motor function of the sympathetic. The irritation thus established would seem also to have a secondary influence upon the sympathetic, resulting in the visceral disturbances that constitute so important a feature of the disease. The well known laws of reflex action suffice to explain the effects of pressure, percussion, etc., and the aberrations of sensation and motility.

The *diagnosis,* after rejecting all cases which fail to present the vertebral tenderness, lies between the affection and the other spinal diseases which, in their earlier stages, may resemble it—chronic myelitis, meningitis, and congestion. An early and correct diagnosis is of the greatest moment as a guide to treatment, the indication for which, in spinal irritation, are quite the reverse of those in the other affections. In a matter of such vital consequence we shall not attempt the imperfect abstract our limit would compel, but refer the reader to the paper itself.

The *prognosis* is favorable, all cases being alleviated by persistent treatment, and nearly all being ultimately cured.

*Treatment.*—The indications are four: "1. To remove the cause; 2. To improve the general tone of the system; 3. To increase the amount of blood in the spinal cord, and improve the nutrition of this organ; 4. To set up a counter-irritant action in the vicinity of the disordered region of the cord." The first indication speaks for itself. The second is met by tonics (as iron, quinine, zinc, cod-liver oil), and especially by alcoholic stimulants. The third by strychnia, phosphorus, phosphoric acid, opium, heat to the spine, the recumbent posture, and, above all, the direct galvanic current, scientifically applied. (The induced current is also of service applied to the affected muscle, where paralysis is present.) Of counter-irritants, blisters and dry cups are to be preferred to antimonial ointment. Wet cups or leeches are inadmissible.

The paper concludes with reports of illustrative cases.

Dr. Austin Flint, Sen., in his remarks to the society at the same time says: None but those whose reminiscences extend back twenty-five or thirty years can appreciate the service rendered to practical medicine by the first writers upon this subject. For up to that time the notion prevailed that nearly every disturbance of the internal organs was the effect of inflammation; all the affections now known as functional were regarded and treated as inflammatory. These writers, by directing the attention of physicians to the spinal cord, and to the employment of tonic and invigorating treatment, did much to change the practice of the day.

At that time, under the name "spinal irritation," or as I choose to call it, "spinal affection," as involving no hypothesis concerning its nature, there seems to fall a group of cases marked by certain features common to all—tenderness of the spinal column (exceedingly common, but not universally present), susceptibility to cold, coldness of extremities, indisposition to physical or mental exertion, and undue fatigue after either, mental depression sometimes amounting to melancholia. These symptoms belong to all the cases, and in addition there were those connected with the various organs. But the name spinal irritation fell more and more into disuse; and, although, for a considerable time, I spoke of spinal affection in my lectures, I at last ceased to do so. Why is this? I recognize to-day the same group of cases I then recognized. But I have been, for some time past, gradually coming to regard these cases as cases of general anæmia, considering the cord to be in a morbid condition, but a condition due to the altered state of the blood. I have been the more confirmed in this view from knowing that profound anæmia may exist and yet present none of its superficial signs: an anæmia patient for example may have a beautiful color in the face. (I think, however, that anæmia, where present, may always be detected in the venous hum.) But, although, I have supposed the anæmia of the cord to be dependent, not upon a deficient supply of blood to that organ, but upon the impoverished character of the blood, yet on this point I should regard Dr. Hammond's opinion as superior to my own.

The adoption of these views has led to a change in my practice. Twenty or thirty years ago I resorted to counter-irritation, especially by means of tartar emetic ointment. I have now come to look back on those days with a feeling of regret that so much needless suffering should have been inflicted. Yet possibly we may be going too far in the opposite direction. Counter-irritation, by dry cups and other of the less severe means may be useful. But, looking upon the essential condition as one of general anæmia, I have found my treatment addressed to this very successful in removing the symptoms of the spinal affection. I have no doubt, however, that the addition of electricity, as proposed by Dr. Hammond, would be of great service. One word as to the chalybeates. I think these remedies often have their values underrated from failure to persist in their use. I think that less depends upon the amount of iron given than upon the length of time for which it is continued; and that, if this be borne in mind, and the chalybeate remedies thoroughly tried, they will be found to give the greatest satisfaction.

